# Yeast Lacking the PP2A Phosphatase Regulatory Subunit Rts1 Sensitizes *rad51* Mutants to Specific DNA Damaging Agents

**DOI:** 10.3389/fgene.2019.01117

**Published:** 2019-11-08

**Authors:** Mustapha Aouida, Abdelmoez Eshrif, Dindial Ramotar

**Affiliations:** ^1^College of Health and Life Sciences, Hamad Bin Khalifa University, Education City, Qatar Foundation, Doha, Qatar; ^2^Maisonneuve-Rosemont Hospital, Research Center, Department of Medicine, Université de Montréal, Montréal, QC, Canada

**Keywords:** anticancer drug bleomycin, mutagenesis, phosphatase subunit, DNA damage, yeast

## Abstract

Rts1 is a regulatory subunit of the trimeric protein phosphatase 2A phosphatase and it participates in many biological processes by modulating the phosphorylation status of proteins. Consistent with its role, mutants lacking Rts1 display multiple phenotypes. We have previously performed a high throughput screen to search for yeast haploid mutants with altered sensitivity to the anticancer drug bleomycin, which acts by damaging the DNA to produce single and double strand breaks. *RTS1* was among the genes that when singly deleted cause sensitivity to bleomycin. We investigate whether Rts1 plays a role in the repair of bleomycin-induced DNA lesions. We show that deletion of the *RTS1* gene in the *rad51* null background, lacking Rad51 known to be involved in the repair of bleomycin-induced DNA lesions, resulted in double mutants that were sensitized to bleomycin and not to other DNA damaging agents that creates DNA adducts. We further show that Rts1 has the ability to bind to DNA and in its absence cells displayed an increase in the frequency of both spontaneous and bleomycin-induced mutations compared to the parent. This is the first report implicating Rts1 with a role in DNA damage and repair, perhaps regulating the phosphorylation status of one or more proteins involved in the repair of DNA strand breaks.

## Introduction

The protein phosphatase 2A (PP2A) is a heterotrimeric complex consisting of a catalytic subunit either Pph21, Pph22, or Pph3, the scaffolding subunit Tpd3, and a regulatory subunit Cdc55 or Rts1. Thus, PP2A can exist in different trimeric forms bearing either the Rts1 (PP2A-Rts1) or the Cdc55 (PP2A-Cdc55) regulatory subunit (Zhao et al., 1997). Rts1 (*Rox Three Suppressor*) was identified as a suppressor of the temperature sensitive allele of the *ROX3* gene, which encodes an essential protein that regulates global stress responses, raising the possibility that Rts1 may also perform a similar role (Evangelista et al., 1996). Several functions have been reported for Rts1 (Eshleman and Morgan, 2014; Petty et al., 2016; Yeasmin et al., 2016; Lucena et al., 2018; Wallis and Nieduszynski, 2018). For example, the overexpression of Rts1 can rescue some of the phenotypes such as genotoxic stress displayed by the absence of the histone acetyltransferase Gcn5. This rescue effect may be related to the coordinated regulation between phosphorylation and acetylation, whereby the phosphorylation of serine 10 promotes acetylation of lysine 14 on histone H3 (Lo et al., 2000). Rts1 has been shown to also control the phosphorylation status of the cell cycle regulated transcription factors Ace2 and Swi5 leading to alteration in the expression of their target genes (Zapata et al., 2014). Cells lacking Rts1 lead to the accumulation of Ace2 in the nucleus of the mother cells, which then activates the expression of the repressor protein Ash1 that blocks for example expression of the HO endonuclease gene (Parnell et al., 2014). In addition, Rts1 is required to promote efficient transcription of the G1 cyclin Cln2, and in the absence of Rts1 cells are unable to modulate the cell size checkpoint, as well as controlling signals in the TORC2 network required for normal control of cell size (Shu et al., 1997; Artiles et al., 2009; Lucena et al., 2018). Moreover, Rts1 is also involved in preventing the activation of the amino acid sensing pathway SPS consisting of the Ssy1 transporter that senses amino acid. Upon stimulation of the SPS pathway, the endoprotease Ssy5 cleaves the transcription factors Stp1 and Stp2 needed to activate expression of the amino acid permeases such as Agp1 and Bap2. In the absence of Rts1, the SPS-pathway constitutively expressed Agp1 and Bap2, indicating that Rts1 can exert control on gene expression (Zhao et al., 1997; Janssens and Goris, 2001; Eckert-Boulet et al., 2006). Thus, Rts1 can influence the functioning of several physiological pathways.

One study examined the *rts1* null mutants for changes in the phosphorylation status of proteins and found that 156 proteins were hyperphosphorylated at 241 sites and another 45 proteins showed decrease in phosphorylation at 59 sites (Zapata et al., 2014). These hyperphosphorylated and dephosphorylated proteins are involved in many biological functions. For example, the hyperphosphorylated proteins Pds1 and Ulp2 are involved in chromosome cohesion, and Swi4 is a transcriptional activator that activates the expression of the late G1 cyclins Cln1 and Cln2 (Dirick and Nasmyth, 1991).

We have previously identified from a high throughput screen of the yeast haploid mutant collection the *RTS1* gene that when deleted cause the resulting *rts1* mutant to be sensitive to the anticancer drug bleomycin (Aouida et al., 2004). Bleomycin is used for treating a limited set of cancers including testicular and lymphomas (Wang and Ramotar, 2002). It acts by damaging the DNA to produce a narrow range of DNA lesions that include single- and double-stranded DNA breaks (Wang and Ramotar, 2002; Ramotar and Wang, 2003). These lesions can be repaired by the homologous recombination DNA repair pathway (Ramotar and Wang, 2003). Mutants lacking proteins in the recombination DNA repair pathway are defective in the repair of DNA strand breaks and are sensitive to bleomycin. In this study, we set out to investigate whether mutants that simultaneously lack Rts1 and one of the recombination DNA repair protein Rad51 would synergistically be more sensitive to bleomycin. We show that (i) deletion of the *RTS1* gene in the *rad51* null background sensitizes the resulting double mutant to bleomycin, but not to DNA damaging agents that creates DNA adducts, (ii) Rts1 is bound to the DNA, and (iii) in the absence of Rts1 the cells have an increase in spontaneous and bleomycin-induced mutations compared to the parent. We propose that Rts1 is required to regulate the phosphorylation status of one or more proteins involved in the repair of DNA strand breaks.

## Methods

### Yeast Strains, Growth Media, and Transformation

The *Saccharomyces cerevisiae* parent and isogenic mutant strains used in this study were all derived from strain BY4741 (*Mat a*, *his3-1*, *leu2-0*, *met15-0*, *ura3-0*) stock of this laboratory. The single and double mutants were created by one-step gene replacement. Yeast cells were grown at 30°C in either yeast extract, peptone, and dextrose (YPD), or minimal synthetic media (SD). Nutritional supplements (20 µg/ml) were added as required (Sherman et al., 1983). The *Escherichia coli* strain DH5α was used for plasmid maintenance and grown in Luria broth. Yeast was transformed by the standard lithium acetate method (Gietz et al., 1995).

### Drug Stocks

All the drugs were purchased from Sigma. Bleomycins, phleomycin, and zeocin were all prepared as stock concentration of 10 mg/ml in water and stored at −20°C. Methyl methanesulfonate and hydrogen peroxide were kept at 4 and −20°C, respectively, and used as the stock concentration. 4-Nitroquinoline-1-oxide was dissolved at 5 mg/ml in dimethyl sulfoxide and kept frozen in aliquots at −20°C.

### Survival Assays and Growth Tests

Survival assays and standard spot tests were performed as previously described (Leduc et al., 2003). Briefly, exponentially growing cells were serially diluted and 5 µl spotted onto solid YPD plates containing the indicated drugs. The plates were photographed after 48 h. In the case of liquid treatment, cells were either teated with bleomycin or zeocin as indicated, serially diluted, plated on YPD solid media and scored for surviving fractions.

### RNA Extraction and Reverse Transcription PCR Analysis

This analysis was performed as previously described (Aouida et al., 2013). The PCR primers used to amplify the *RTS1* gene were RT-PCR-RTS1-F: ACATCAAAGAAGCCCGCTTCGGCT​AGTAGCT CTTC and RT-PCR-RTS1-R: GATCTCGGAATGTC​GATACTTTTGTGAGGAGGCGT. The *ACT1* gene was used as a control (Aouida et al., 2013).

### Mutagenesis Assay

Spontaneous and drug-induced mutations were performed as previously described (Shatilla et al., 2005). Briefly, 15 to 20 single colonies were each grown in 1 ml of −Arg drop out liquid minimal media for 24 h. In the case of treatment, the cells were treated with the indicated drugs, washed, and resuspended in fresh media. The cultures 100 µl were each diluted and plated onto the same media with agar to calculate the number of viable cells. The remaining 900 µl were spun for 30 s at 10,000 rpm in an Eppendorf centrifuge and the pellets were resuspended and plated onto −Arg drop out solid media containing 25 µg/ml of L-canavanine. Individual colonies that appeared after 5 days of growth were considered to be canavanine resistant, Can^R^. The frequency of mutations were calculated using data from three to five independent experiments whereby the total number of Can^R^ colonies were divided by the total number of colonies from the −Arg plates.

### RADAR Assay

This assay was adapted for studies with yeast cells (Kiianitsa and Maizels, 2014). Cells were grown in 1 ml of YPD media and incubated at 30°C overnight. Next day, the cells were subcultured for 2 to 3 h. An aliquot of 100 µl of the subcultured cells was pelleted and resuspended in 150 µl of 100 mM PIPES/KOH pH 9.4 containing 10 mM of dithiothreitol. The cells were incubated 10 min at 30°C, pelleted and resuspended in 250 µl of YPD containing 0.6 M sorbitol, 25 mM Tris–HCl pH 7.5, and 50 µl of lyticase (5 mg/ml). The cells were incubated for 30 min at 30°C, then washed twice with 150 µl of YPD containing 0.6 M sorbitol and 25 mM of Tris–HCl pH 7.5. To the cell pellets, 250 µl of MB (4 M guanine thiocyanate, 10 mM Tris–HCL pH 6.5, 20 mM EDTA, 4% Triton X100, 1% Sarkosyl (sodium lauroyl sarcosinate), and 1% dithiothreitol) and 125 µl of ethanol (EtOH) 100% was added, then stored at −20°C for 5 min, centrifuged for 15 min at max speed and the recovered pellet containing the DNA and bound proteins was washed twice with 200 µl of EtOH 75%, each time with centrifugation at max speed for 10 min. The protein-bound DNA pellet was resuspended in 200 µl 8 mM of NaOH and an aliquot of 100 µl was diluted in 200 µl of TBS (50 mM Tris–HCl pH 7.5 and 150 mM NaCl). A sample of 10 µl was used for DNA quantification using Syber Green I Dye and fixed amounts (or different amounts) of the DNA were loaded onto a slot blot equipped with nitrocellulose membrane. Empty wells were loaded with TBS containing bromophenol blue and a gentle vacuum was applied to the slot blot apparatus and once the wells were empty, each well was washed once with 200 µl of TBS and the recovered membrane was cut and each piece was processed by Western blot analysis with the appropriate antibodies (Kiianitsa and Maizels, 2014).

### Plasmid Construction

The entire *RTS1* gene was cloned into a centromeric plasmid YCplac-111 and a multicopy plasmid YEplac-185 and introduced into the *rts1Δ* mutant. Both plasmids pYCp111-RTS1 and pYEplac181-RTS1 were created by gap repair using the primers RTS1-GAP-F1: GAAACAGCTATGACCATGG​ATTACGCCAAG CTTGCATGCCTG and RTS1-GAP-R1: ATTAAGTTGGGTAACGCCAGGG TTTTCCCAGTCACGAC​GTT. The plasmid DNA carrying the *RTS1* gene under the expression of its own promoter was recovered from the yeast cells by glass bead extraction, transformed into *E. coli* cells and verified by DNA sequence analysis using the primers RTS1-F and RTS1-R: CGCTTTGTTTTCCACTTCAATTGGTAGGC and CGGGGATTCTATCTTTGGTTTCTTCAACAAG, respectively.

## Results

### Deficiency in Rts1 Sensitizes *rad51Δ* Mutant to the DNA Damaging Agent Bleomycin

**Figure 1:**
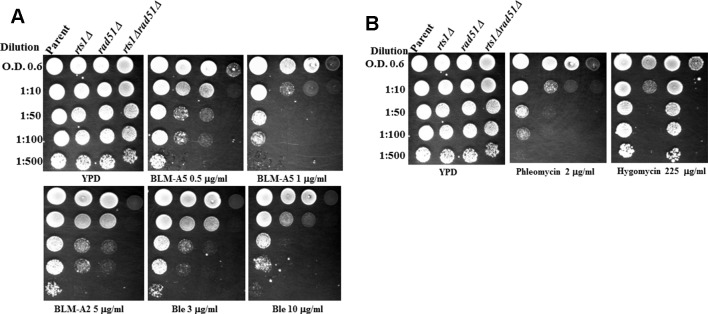
Deletion of the *RTS1* gene sensitizes the *rad51Δ* mutant to bleomycin and phleomycin. **(A)** Spot test analysis showing the sensitivity of the single and double mutants towards bleomycin. Overnight cultures of the indicated strains grown in yeast extract, peptone, and dextrose (YPD) media were adjusted to OD 600 nm of 0.6, serially diluted and spotted onto plates without and with the indicated concentration of the drugs. **(B)** Spot test analysis showing the sensitivity of the single and double mutants towards phleomycin and hygromycin. The experiment was performed as Figure 1A. The plates were incubated for 48 h at 30°C before photographed. The results are represented of three independent experiments.

*rts1Δ* mutants have been reported to display several phenotypes which include slow growth phenotype and sensitivity towards the protein synthesis inhibitor hygromycin B (Shu et al., 1997; Barreto et al., 2011; Arino et al., 2019). Since we have previously identified from a large scale screen that the *RTS1* gene when deleted caused sensitivity to the DNA strand-breaking agent bleomycin (Aouida et al., 2004), we decided to examine more closely whether *rts1Δ* mutant would have a defect in DNA repair. To initiate this work, we recreated isogenic strains from the parental BY4741 background, which was used to generate the haploid collection of single gene-deletion mutants. These isogenic strains included *rts1Δ*, *rad51Δ* deleted for *RAD51* gene that encodes the Rad51 protein known to be involved in the repair of DNA double strand breaks, and the double mutant *rts1Δ rad51Δ*. Cultures of these strains were grown overnight, serially diluted, and then spotted onto solid rich media plates containing the indicated drugs (Figure 1A). The spot analysis revealed that the *rts1Δ* mutant was indeed sensitive to bleomycin (Figure 1A). Likewise, the *rts1Δ* mutant was sensitive to phleomycin often referred to as zeocin (Figure 1B), a DNA damaging agent that is also known to attack and destroy the DNA to create DNA strand breaks. We confirmed that the *rts1Δ* mutant was sensitive to hygromycin B, as previously reported by Barreto et al., 2011, but not the *rad51Δ* mutant that is known to be sensitive to agents that create DNA double strand breaks (Figure 1B) (Barreto et al., 2011). It would appear that Rts1 performs distinct functions in the detoxification of hygromycin B and combating the genotoxic effects of bleomycin.

Since bleomycin is known to create a variety of DNA lesions such as oxidized AP sites, DNA single strand breaks terminated with blocked 3′-ends, as well as DNA double strand breaks, we checked whether *rts1Δ* mutant would display the same phenotypes as a mutant *rad51Δ* that is defective in DNA double strand break repair. Both the single *rts1Δ* and *rad51Δ* mutants were sensitive to the various forms of bleomycins [bleomycin A5, bleomycin A2, and blenoxane (Ble)], however, the *rad51Δ* mutant was more sensitive than the *rts1Δ* mutant (Figure 1A). In contrast, the double *rts1Δ rad51Δ* mutant displayed an increased sensitivity to bleomycin, which was more conspicuous at lower concentration of the drug (Figure 1A). The experiment was performed in a different manner by treating cells in liquid cultures either with increasing concentrations of bleomycin or a fixed concentration over time and then scored for the surviving fractions (Leduc et al., 2003; Aouida et al., 2004). The result of this analysis revealed that the *rad51Δ* mutant was significantly more sensitive to bleomycin than the *rts1Δ* mutant, and that the double mutant was extremely sensitive consistent with the data from the spot test assay (**Figures S1A, B**). We interpret these findings to suggest that Rts1 may perform an independent role from Rad51 in processing bleomycin-induced DNA lesion.

### Rts1 Deletion Sensitizes *rad51Δ* Mutant to Hydrogen Peroxide

**Figure 2:**
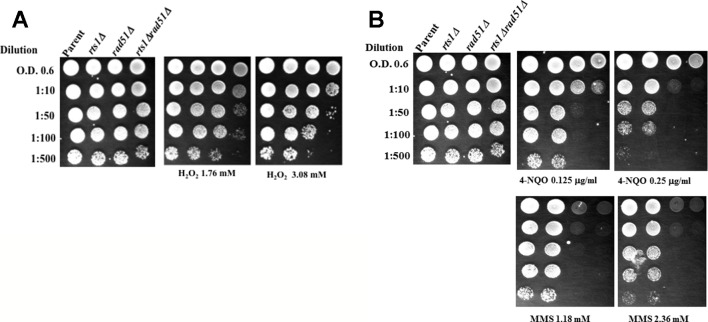
Deletion of the *RTS1* gene sensitizes the *rad51Δ* mutant to H_2_O_2_, but not to 4-nitroquinoline-1-oxide or methyl methanesulfonate. **(A**, **B)** Spot test analyses were performed as in **Figure 1**. 4-Nitroquinoline-1-oxide (4-NQO), methyl methanesulfonate (MMS). The results are representative of three independent experiments.

We examined whether the *rts1Δ* mutant would be sensitive to other types of DNA damaging agents. The *rts1Δ* mutant alone did not show any striking sensitivity to other agents that can produce DNA strand breaks such as hydrogen peroxide (H_2_O_2_) (**Figure 2A**). However, when *RTS1* was deleted in combination with the *RAD51* gene, the resulting double *rts1Δ rad51Δ* mutant was sensitive to H_2_O_2_ as compared to the single *rad51Δ* mutant. Deletion of the *RTS1* gene did not appear to sensitize the *rad51Δ* mutant to 4-nitroquinoline-1-oxide, an agent that creates bulky DNA adducts or to the alkylation agent methyl methane sulfonate that indirectly creates apurinic/apyrimidinic (AP) sites in the genome (**Figure 2B**). However, the double *rts1Δ rad51Δ* mutant seemed to grow slightly slower on the 4-nitroquinoline-1-oxide plates (**Figure 2B**). Collectively, the data suggest that Rts1 may play a role in processing specific types of DNA lesions such as DNA strand breaks that are induced by bleomycin and hydrogen peroxide. Neither 4-nitroquinoline-1-oxide or methyl methane sulfonate directly creates DNA strand breaks, but can block DNA replication leading to DNA strand breaks that trigger recombination.

### Expression of the *RTS1* Gene Rescues the *Rts1Δ* Phenotypes.

**Figure 3:**
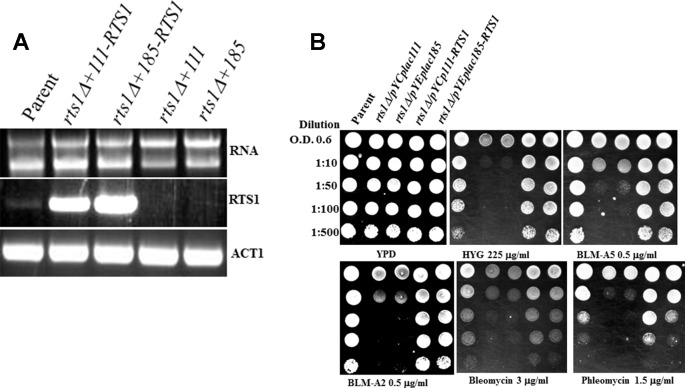
Expression of the *RTS1* gene rescues the phenotypes of the *rts1Δ* mutant. **(A)** Reverse transcription PCR (RT-PCR) showing expression of the *RTS1* gene. The entire *RTS1* gene including its promoter was subcloned into the single copy and multicopy vectors YCplac-111 and YEplac-185, respectively and introduced into the *rts1Δ* mutant. **(B)** Spot test analysis. The cells were spotted onto the indicated drug plates as in **Figure 1**. The results are representative of three independent experiments.

To ensure that the phenotypes observed were due to the *RTS1* gene deletion, we reintroduced the *RTS1* gene into the *rts1Δ* mutant and scored for sensitivity to both hygromycin and bleomycin using spot test analysis. The entire *RTS1* gene was cloned into a centromeric vector YCplac-111 and a multicopy vector YEplac-185 and introduced into the *rts1Δ* mutant. Both plasmids p111-RTS1 and p181-RTS1 expressed the *RTS1* gene as determined by reverse transcription PCR using gene specific primers (**Figure 3A**). *RTS1* expression was significantly higher from the plasmids as opposed to the endogenous level from the parent strain (**Figure 3A**, lanes 2 and 3 vs. lane 1). The expressed *RTS1* restored parental level of hygromycin and bleomycin resistance to the *rts1Δ* mutant, but not when the mutant carried the empty vectors, YCplac-111 or YEplac-185 (**Figure 3B**). We observed that the overexpression of *RTS1* did not provide additional resistance to these and other drugs (Figure 3B), suggesting that Rts1 is not a limiting factor in the cells. Altogether, the phenotypes we observed are the result of the deleted *RTS1* gene.

### Rts1 Binds to the Chromatin, but Its Level Is Unaffected by Drug Treatment

**Figure 4:**
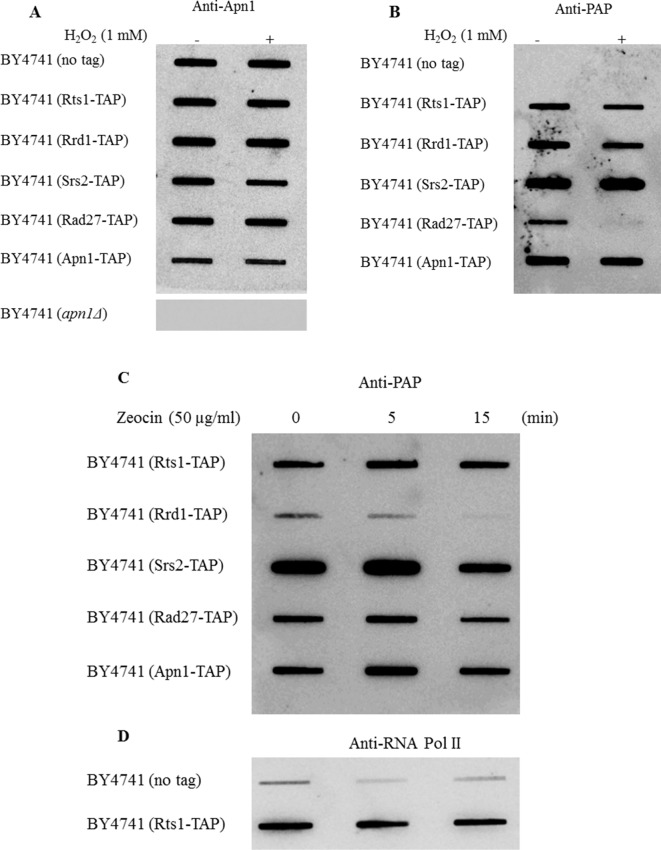
Rts1-TAP is bound to the chromatin similar to the Tandem Affinity Purification (TAP) tagged DNA binding proteins Srs2, Rad27, and Apn1. **(A**,** B)** The indicated strains were untreated and treated with H_2_O_2_ (1 mM for 30 min) and 700 ng of the protein bound DNA from each sample (see *RADAR Assay*) was loaded onto a slot blot and assess for the binding of the indicated nuclear proteins using antibodies against Apn1 and the TAP tag (anti-PAP), respectively. For the anti-Apn1 control, the *apn1Δ* mutant was used and for the anti-PAP control the parent strain BY4741 was used that lacks an endogenous TAP tag. **(C**,** D)** Cells were treated with the phleomycin analogue zeocin and processed by the RADAR assay. The blots were probed with anti-PAP **(C)** and anti-RNA polymerase II **(D)**. The data are representative of three independent experiments.

Rts1 has been previously shown to localize to the nucleus during the cell cycle and bound to the centromere (Eshleman and Morgan, 2014). We used a different approach to test if Rts1 is bound to the chromatin and more importantly to determine if it is affected by drug treatment using the rapid approach to DNA adduct recovery (RADAR) assay (Kiianitsa and Maizels, 2014). In this assay, guanine thiocyanate is used to trap chromatin-bound proteins, which are then processed by a slot blot for detection by immunoblot analysis using specific antibodies. For this experiment, we prepared chromatin-bound proteins from the parent and the indicated strains that were tagged with the Tandem Affinity Purification (TAP) tag at the endogenous locus of known genes encoding DNA binding proteins. This was necessary as antibodies against the proteins tested were not available from commercial sources. In the control experiment, all the strains showed the presence of the DNA repair protein Apn1 on the chromatin, but not in the strain deleted for the *APN1* gene, i.e., BY4741 (*apn1Δ*) (**Figure 4**)(Ramotar et al., 1993). Treatment with H_2_O_2_ (1 mM for 30 min) did not affect the level of Apn1 detected in each of the strains (**Figure 4A**). When these same samples were probed with anti-PAP, which can detect the protein A portion of the TAP tag, all the strains showed the respective proteins (Rts1-, Rrd1-, Srs2-, Rad27-, and Apn1-TAP) bound to the chromatin (**Figure 4B**). No protein band was detected in the parent strain lacking the TAP tag (**Figure 4B**). Since Rts1 is detected on the chromatin bound protein in a manner similar to the known DNA binding proteins (Srs2, Rad27, and Apn1), we suggest that Rts1 is a DNA binding protein. Neither Rts1-, Rrd1-, Srs2-, or Apn1-TAP showed any significant loss of binding to the chromatin upon treatment with H_2_O_2_. However, Rad27-TAP was the only protein that displayed significant loss from the chromatin when challenged with H_2_O_2_ (**Figure 4B**).

Besides H_2_O_2_, we also treated the strains with zeocin, an analogue of one of the forms of phleomycins, namely phleomycin D1, which creates DNA double strand breaks. While all the strains were sensitive to the increasing dose of zeocin, the parent was the least sensitive followed by *rts1Δ*, *rad51Δ*, and the *rts1Δ rad51Δ* double mutant (**Figure S2**). Upon treatment with zeocin, we observed that only the transcription elongation factor Rrd1-TAP showed significant loss from the chromatin, and not Rts1 or the other proteins (**Figure 4C**). It is noteworthy that unlike the effect of H_2_O_2_ on the loss of chromatin-bound Rad27-TAP, zeocin had no such effect on this protein (**Figures 4B vs. C**). For the zeocin treatment, BY4741 (no tag) was used as a control, but probed for anti-RNA polymerase II (**Figure 4D**). As in the case of all immunoglobulin G derived antibodies such as anti-RNA pol II, they have the ability to recognize the protein A portion of the TAP tag (**Figure 4D**). These data suggest that (i) Rts1 is capable of binding to DNA and (ii) its level is not influenced by agents that can create single or double strand breaks.

### Rts1 Deficiency Elevates Both the Spontaneous and Bleomycin-Induced Mutations

From the above findings, we postulate that the sensitivity of the *rts1Δ* mutant to bleomycin might be related to a defect in DNA repair. Defects in DNA repair are known to be associated with elevated mutations (Masson and Ramotar, 1996; Shatilla and Ramotar, 2002). As such, we tested whether *rts1Δ* mutant would exhibit an increased frequency of mutations. To do this experiment, we cultured cells and scored for resistance to the arginine analog canavanine. Canavanine resistance can occur as a result of mutation in the *CAN1* gene, encoding the arginine transporter, which prevents the uptake of canavanine (Shatilla et al., 2005). Using this analysis, we observed that the *rts1Δ* mutant displayed a 3-fold increase in the frequency of spontaneous *Can* *^R^* colonies as compared to the parent, while the DNA repair defected *rad51Δ* mutant showed 30-fold increase (**Table 1**). The double mutant deleted for both the *RTS1* and *RAD51* genes accumulated substantially higher levels of *Can* *^R^* mutations, > 60-fold (**Table 1**), strongly suggesting that Rts1 plays a role in suppressing spontaneous mutations.

**Table 1:** Frequency of spontaneous and induced Can^R^ mutations (×10^−^^7^).

Strains	Untreated	BLM-treated (1 µg/ml/1 h)	MMS-treated (0.1%/1 h)	Fold increased with BLM
Parent	2.31 ± 0.6	8.4 ± 2.5	11.32 ± 0.7	3.6
*rts1Δ*	8.79 ± 0.4	98.1 ± 4.7	12.76 ± 1.1	11.2
*rad51Δ*	69.33 ± 4.5	123.9 ± 9.5	ND	1.8
*rts1Δrad51Δ*	149.43 ± 13.2	425.0 ± 23.2	ND	2.9
Parent/pYCplac111	2.63 ± 0.7	9.1 ± 1.3		3.5
Parent/pYCplac111-RTS1	2.70 ± 0.3	8.1 ± 0.9		3.0
*rts1Δ*/pYCplac111	9.4 ± 0.6	101.2 ± 3.7		10.8
*rts1Δ*/pYCplac111-RTS1	2.6 ± 0.2	8.8 ± 1.8		3.4

The mutation frequency was calculated using 15 to 20 independent colonies from untreated and drug treatment from three to five separate experiments. The fold increased in the bleomycin-induced mutation frequency was relative to the untreated strain.

We next examined the effect of bleomycin treatment on the frequency of *Can* *^R^* mutations. Unlike methyl methane sulfonate, bleomycin treatment induced a higher level of mutations, 11-fold in the *rts1Δ* mutant as compared to the 3-fold observed for the parent (**Table 1**). This higher level of bleomycin-induced mutations in the *rts1Δ* mutant was suppressed upon introduction of the plasmid pYCplac111-RTS1, but not by the vector pYCplac111 (**Table 1**). More importantly, substantially higher levels of bleomycin-induced mutations (over 50-fold) were found in the *rts1Δ rad51Δ* double mutant, as compared to the single *rts1Δ* (11-fold) and *rad51Δ* (15-fold) mutants relative to the levels observed with the bleomycin treated parent (**Table 1**). Thus, it would appear that Rts1 is required to repair specific spontaneous DNA lesions, which are likely to be the same ones induced by bleomycin.

## Discussion

We previously identified the *RTS1* gene when deleted display sensitivity to the anticancer drug bleomycin, which acts by attacking the DNA to generate both single and double strand breaks (Ramotar and Wang, 2003). We set out to ask whether Rts1 has a role in DNA repair. Our approach involved deleting the *RTS1* gene in combination with a defect in recombinational DNA repair, which is the major DNA repair pathway that can process bleomycin-induced DNA strand breaks (Rulten and Grundy, 2017; Kim and Mirkin, 2018). We showed that deleting the *RTS1* gene in the *rad51Δ* recombinational defective mutant sensitized the *rts1Δ rad51Δ* double mutant to bleomycin, raising the possibility that Rts1 may perform a distinct function to prevent the genotoxic effects of the drug. The observation that these *rts1Δ rad51Δ* double mutants were hypersensitive to the single strand break-generating agent H_2_O_2_, as compared to the *rad51Δ* single mutant further supports the notion that Rts1 could be involved in the repair of DNA strand breaks. Rts1 did not sensitize the *rad51Δ* mutants to 4-nitroquinoline-1-oxide, which creates bulky DNA lesions, suggesting that if Rts1 is involved in DNA repair its role might be restricted to specific types of DNA lesions such as DNA strand breaks.

Many proteins that are involved in DNA repair have been shown to bind to DNA and in the absence of these proteins, the cells display elevated levels of mutations (Shatilla and Ramotar, 2002;Tounekti et al., 2006). We used the RADAR assay, which has been established to detect proteins bound to DNA (Kiianitsa and Maizels, 2014). Indeed, the assay revealed that Rts1 was bound to the DNA, as seen for known DNA repair proteins such as the helicase Srs2 and the AP endonuclease Apn1 that bind to DNA. While some DNA repair proteins can disassociate from the DNA in response to DNA damage such as Rad27 in response to H_2_O_2_, Rts1 was not affected by treatment with either H_2_O_2_ or zeocin in a manner similar to Srs2 or Apn1. Thus, it would appear that the function of Rts1 is constantly needed on the chromatin.

A compelling evidence for Rts1 involvement in DNA repair to maintain genomic stability comes from the observation that the null mutant displayed 3.8-fold increase in the frequency of spontaneous mutation, as compared to the parent. Importantly, the observation that the mutation frequency was stimulated nearly 11-fold in the *rts1Δ* mutant upon treatment with bleomycin, as compared to 3.6 fold in the parent, strongly suggests that Rts1 is involved in some aspect in the processing of bleomycin-induced DNA lesions (**Table 1**). Of note, MMS treatment did not enhance the frequency of mutations in the *rts1Δ* mutant as compared to the parent, consistent with Rts1 performing a role in processing specific types of DNA lesions. While MMS creates alkylated bases that are repaired by the base excision repair pathway, bleomycin generates primarily single- and double-strand breaks that require processing by the homologous and nonhomologous DNA repair pathways.

What could be the function of Rts1 in processing bleomycin-induced DNA lesions? Processing of bleomycin-induced double strand breaks by the homologous recombination pathway requires multiple steps. The double strand breaks must be first resected in the 5′ to 3′- direction by a complex of proteins that include Mre11-Rad50-Xrs2 to create a long ssDNA tail, and this is aided by the actions of the DNA helicase Sgs1, the 5′ to 3′ exonuclease Exo1 and an enzyme DNA2 with 5′-flap endonuclease and ATP-dependent helicase activities. As the resection occurs, the 3′ single stranded DNA tail is coated by the single strand DNA binding protein replication protein A (RPA), which is phosphorylated. The single stranded DNA-bound with RPA is replaced by the recombinase Rad51 that is assisted by mediator proteins Rad54, Rad55, and Rad57. Once Rad51 is loaded it facilitates the loading of more Rad51 and this displaces the RPA that is on the single stranded DNA tail. The Rad51 coated single stranded DNA invades a sister chromatid or homologous duplex DNA molecule and performs a search for homology. Invasion and DNA strand exchange occurs with the duplex DNA. DNA synthesis takes place, followed by resolution and finally ligation. During these multiple steps many proteins are phosphorylated and these must be dephosphorylated to allow the subsequent reactions. For example, phosphorylation of RPA has recently been shown to suppress the resection step and serves as a feedback loop to promote the strand invasion activity (Soniat et al., 2019). Besides RPA, there are many other proteins in the homologous recombination pathway that are phosphorylated such as Rad54, Rad55, and Rad57. In fact, a mass spectrometry study by Zapata et al., 2014 identified many proteins including the DNA resection proteins Xrs2 and DNA2, as well as Srs2 which stimulates Rad51, with upregulated phosphorylation sites in the *rts1Δ* mutant (Dupaigne et al., 2008; Le Breton et al., 2008; Zapata et al., 2014; Soniat et al., 2019).

In addition to the components that are regulated by phosphorylation/dephosphorylation in the homologous recombination pathways there are factors that are also phosphorylated in the nonhomologous end joining pathway that also functions to repair DNA double strand breaks. Some of these factors include Ku70 and Ku80 that bind to the ends of DNA double strand breaks to recruit a kinase that phosphorylates Ku70/80. Both Ku70/80 proteins are removed from the ends of the DNA *via* dephosphorylation. The previously reported observation that cells devoid of the Ku proteins are sensitive to bleomycin and that these proteins require dephosphorylation for their turnover also suggest a possible function for Rts1.

Could Rts1 act as a DNA 3′-phosphodiesterase? Several proteins are known to have phosphodiesterase activity such as Tpp1 that can remove phosphate groups left at the 3′-ends of DNA caused by bleomycin and H_2_O_2_. We note that Rts1 does not share significant identity with Tpp1 and thus it is unlikely to also possess a phosphodiesterase activity that would be involved in the direct removal of phosphate at the 3′-ends of damaged DNA. We further note that the mammalian counterpart B56 of the yeast Rts1 is believed to play a role in tumor suppression (Yang and Phiel, 2010). It is involved in both anti- and proapoptotic processes such as p53-dependent apoptosis (Jin et al., 2010).

In short, we provide the first evidence that Rts1 might play a role in mediating the repair of DNA strand breaks, particularly those created by bleomycin. This role would require that Rts1 removes phosphate groups to either activate or inactive proteins in the DNA strand break repair pathways. Since there are several biochemical steps involved in the repair of DNA strand breaks, further experiments will be needed to test which of the proteins in these steps must be dephosphorylated to promote efficient repair of the DNA strand breaks.

## Data Availability Statement

All datasets generated for this study are included in the article/Supplementary Material.

## Author Contributions

MA and DR conceived the ideas, MA and AE conducted the experiments, MA, AE, and DR analyzed the results, MA and DR wrote the paper.

## Funding

This work was supported by the Canadian Institute of Health Research, under Grant PJT-153141 to DR. The publication cost was paid by Hamad Bin Khalifa University, Qatar Foundation.

## Conflict of Interest

The authors declare that the research was conducted in the absence of any commercial or financial relationships that could be construed as a potential conflict of interest.

## References

[B1] Aouida M. Page N. Leduc A. Peter M. Ramotar D. (2004). A genome-wide screen in *Saccharomyces cerevisiae* reveals altered transport as a mechanism of resistance to the anticancer drug bleomycin. Cancer Res. 64, 1102–1109. 10.1158/0008-5472.CAN-03-2729 14871844

[B2] Aouida M. Rubio-Texeira M. Thevelein J. M. Poulin R. Ramotar D. (2013). Agp2, a member of the yeast amino acid permease family, positively regulates polyamine transport at the transcriptional level. PloS One 8, e65717. 10.1371/annotation/ff0ad3b6-fff4-4783-8c2e-968a95283ab8 23755272 PMC3670898

[B3] Arino J. Velazquez D. Casamayor A. (2019). Ser/Thr protein phosphatases in fungi: structure, regulation and function. Microb. Cell 6, 217–256. 10.15698/mic2019.05.677 31114794 PMC6506691

[B4] Artiles K. Anastasia S. Mccusker D. Kellogg D. R. (2009). The Rts1 regulatory subunit of protein phosphatase 2A is required for control of G1 cyclin transcription and nutrient modulation of cell size. PloS Genet. 5, e1000727. 10.1371/journal.pgen.1000727 19911052 PMC2770260

[B5] Barreto L. Canadell D. Petrezselyova S. Navarrete C. Maresova L. Perez-Valle J. (2011). A genomewide screen for tolerance to cationic drugs reveals genes important for potassium homeostasis in *Saccharomyces cerevisiae*. Eukaryot Cell 10, 1241–1250. 10.1128/EC.05029-11 21724935 PMC3187046

[B6] Dirick L. Nasmyth K. (1991). Positive feedback in the activation of G1 cyclins in yeast. Nature 351, 754–757. 10.1038/351754a0 1829507

[B7] Dupaigne P. Le Breton C. Fabre F. Gangloff S. Le Cam E. Veaute X. (2008). The Srs2 helicase activity is stimulated by Rad51 filaments on dsDNA: implications for crossover incidence during mitotic recombination. Mol. Cell 29, 243–254. 10.1016/j.molcel.2007.11.033 18243118

[B8] Eckert-Boulet N. Larsson K. Wu B. Poulsen P. Regenberg B. Nielsen J. (2006). Deletion of RTS1, encoding a regulatory subunit of protein phosphatase 2A, results in constitutive amino acid signaling via increased Stp1p processing. Eukaryot Cell 5, 174–179. 10.1128/EC.5.1.174-179.2006 16400180 PMC1360261

[B9] Eshleman H. D. Morgan D. O. (2014). Sgo1 recruits PP2A to chromosomes to ensure sister chromatid bi-orientation during mitosis. J. Cell Sci. 127, 4974–4983. 10.1242/jcs.161273 25236599 PMC4231310

[B10] Evangelista C. C. Rodriguez Torres A. M. Limbach M. P. Zitomer R. S. (1996). Rox3 and Rts1 function in the global stress response pathway in baker's yeast. Genetics 142, 1083–1093. 8846889 10.1093/genetics/142.4.1083 PMC1207109

[B11] Gietz R. D. Schiestl R. H. Willems A. R. Woods R. A. (1995). Studies on the transformation of intact yeast cells by the LiAc/SS-DNA/PEG procedure. Yeast 11, 355–360. 10.1002/yea.320110408 7785336

[B12] Janssens V. Goris J. (2001). Protein phosphatase 2A: a highly regulated family of serine/threonine phosphatases implicated in cell growth and signalling. Biochem. J. 353, 417–439. 10.1042/bj3530417 11171037 PMC1221586

[B13] Jin Z. Wallace L. Harper S. Q. Yang J. (2010). PP2A:B56{epsilon}, a substrate of caspase-3, regulates p53-dependent and p53-independent apoptosis during development. J. Biol. Chem. 285, 34493–34502. 10.1074/jbc.M110.169581 20807766 PMC2966064

[B14] Kiianitsa K. Maizels N. (2014). Ultrasensitive isolation, identification and quantification of DNA-protein adducts by ELISA-based RADAR assay. Nucleic Acids Res. 42, e108. 10.1093/nar/gku490 24914050 PMC4117749

[B15] Kim K. P. Mirkin E. V. (2018). So similar yet so different: the two ends of a double strand break. Mutat. Res. 809, 70–80. 10.1016/j.mrfmmm.2017.06.007 28693746

[B16] Le Breton C. Dupaigne P. Robert T. Le Cam E. Gangloff S. Fabre F. (2008). Srs2 removes deadly recombination intermediates independently of its interaction with SUMO-modified PCNA. Nucleic Acids Res. 36, 4964–4974. 10.1093/nar/gkn441 18658248 PMC2528196

[B17] Leduc A. He C. H. Ramotar D. (2003). Disruption of the *Saccharomyces cerevisiae* cell-wall pathway gene SLG1 causes hypersensitivity to the antitumor drug bleomycin. Mol. Gen. Genomics 269, 78–89. 10.1007/s00438-003-0812-8 12715156

[B18] Lo W. S. Trievel R. C. Rojas J. R. Duggan L. Hsu J. Y. Allis C. D. (2000). Phosphorylation of serine 10 in histone H3 is functionally linked in vitro and in vivo to Gcn5-mediated acetylation at lysine 14 . Mol. Cell 5, 917–926. 10.1016/S1097-2765(00)80257-9 10911986

[B19] Lucena R. Alcaide-Gavilan M. Schubert K. He M. Domnauer M. G. Marquer C. (2018). Cell size and growth rate are modulated by torc2-dependent signals. Curr. Biol. 28, 196–210. 10.1016/j.cub.2017.11.069 29290562 PMC5787035

[B20] Masson J. Y. Ramotar D. (1996). The *Saccharomyces cerevisiae* IMP2 gene encodes a transcriptional activator that mediates protection against DNA damage caused by bleomycin and other oxidants. Mol. Cell Biol. 16, 2091–2100. 10.1128/MCB.16.5.2091 8628275 PMC231196

[B21] Parnell E. J. Yu Y. Lucena R. Yoon Y. Bai L. Kellogg D. R. (2014). The Rts1 regulatory subunit of PP2A phosphatase controls expression of the HO endonuclease via localization of the Ace2 transcription factor. J. Biol. Chem. 289, 35431–35437. 10.1074/jbc.M114.611715 25352596 PMC4271228

[B22] Petty E. L. Lafon A. Tomlinson S. L. Mendelsohn B. A. Pillus L. (2016). Promotion of cell viability and histone gene expression by the acetyltransferase Gcn5 and the protein phosphatase PP2a in *Saccharomyces cerevisiae*. Genetics 203, 1693–1707. 10.1534/genetics.116.189506 27317677 PMC4981271

[B23] Ramotar D. Kim C. Lillis R. Demple B. (1993). Intracellular localization of the Apn1 DNA repair enzyme of *Saccharomyces cerevisiae*. Nuclear transport signals and biological role. J. Biol. Chem. 268, 20533–20539. 7690756

[B24] Ramotar D. Wang H. (2003). Protective mechanisms against the antitumor agent bleomycin: lessons from *Saccharomyces cerevisiae*. Curr. Genet. 43, 213–224. 10.1007/s00294-003-0396-1 12698269

[B25] Rulten S. L. Grundy G. J. (2017). Non-homologous end joining: common interaction sites and exchange of multiple factors in the DNA repair process. Bioessays 39, 1–2. 10.1002/bies.201600209 28133776

[B26] Shatilla A. Leduc A. Yang X. Ramotar D. (2005). Identification of two apurinic/apyrimidinic endonucleases from Caenorhabditis elegans by cross-species complementation. DNA Repair (Amst) 4, 655–670. 10.1016/j.dnarep.2005.02.005 15907773

[B27] Shatilla A. Ramotar D. (2002). Embryonic extracts derived from the nematode Caenorhabditis elegans remove uracil from DNA by the sequential action of uracil-DNA glycosylase and AP (apurinic/apyrimidinic) endonuclease. Biochem. J. 365, 547–553. 10.1042/bj20020375 11966472 PMC1222696

[B28] Sherman, F, Fink, G, and Hicks, J, editors. (1983). Methods in yeast genetics. New York: Cold Spring Harbor.

[B29] Shu Y. Yang H. Hallberg E. Hallberg R. (1997). Molecular genetic analysis of Rts1p, a B' regulatory subunit of *Saccharomyces cerevisiae* protein phosphatase 2A. Mol. Cell Biol. 17, 3242–3253. 10.1128/MCB.17.6.3242 9154823 PMC232177

[B30] Soniat M. M. Myler L. R. Kuo H. C. Paull T. T. Finkelstein I. J. (2019). RPA phosphorylation inhibits DNA resection. Mol. Cell. 75 (1), 145–153. 10.1016/j.molcel.2019.05.005 31153714 PMC6625828

[B31] Tounekti K. Aouida M. Leduc A. Yang X. Poschmann J. Belhadj O. (2006). Deletion of the chromatin remodeling gene SPT10 sensitizes yeast cells to a subclass of DNA-damaging agents. Environ. Mol. Mutagenesis. 47 (9), 707–717. 10.1002/em20260 17078097

[B32] Wallis A. B. A. Nieduszynski C. A. (2018). Investigating the role of Rts1 in DNA replication initiation. Wellcome Open Res. 3, 23. 10.12688/wellcomeopenres.13884.1 29721551 PMC5897792

[B33] Wang H. Ramotar D. (2002). Cellular resistance to bleomycin in *Saccharomyces cerevisiae* is not affected by changes in bleomycin hydrolase levels. Biochem. Cell Biol. 80, 789–796. 10.1139/o02-167 12555812

[B34] Yang J. Phiel C. (2010). Functions of B56-containing PP2As in major developmental and cancer signaling pathways. Life Sci. 87, 659–666. 10.1016/j.lfs.2010.10.003 20934435 PMC2993835

[B35] Yeasmin A. M. Waliullah T. M. Kondo A. Kaneko A. Koike N. Ushimaru T. (2016). Orchestrated action of PP2a antagonizes atg13 phosphorylation and promotes autophagy after the inactivation of TORC1 . PloS One 11, e0166636. 10.1371/journal.pone.0166636 27973551 PMC5156417

[B36] Zapata J. Dephoure N. Macdonough T. Yu Y. Parnell E. J. Mooring M. (2014). PP2ARts1 is a master regulator of pathways that control cell size. J. Cell Biol. 204, 359–376. 10.1083/jcb.201309119 24493588 PMC3912523

[B37] Zhao Y. Boguslawski G. Zitomer R. S. Depaoli-Roach A. A. (1997). *Saccharomyces cerevisiae* homologs of mammalian B and B' subunits of protein phosphatase 2A direct the enzyme to distinct cellular functions. J. Biol. Chem. 272, 8256–8262. 10.1074/jbc.272.13.8256 9079645

