# Supplementary material for: Yeast Lacking the PP2A Phosphatase Regulatory Subunit Rts1 Sensitizes rad51 Mutants to Specific DNA Damaging Agents
Source: Front Genet. 2019 Nov 8;10:1117. doi: 10.3389/fgene.2019.01117 (PMC6857479; doi:10.3389/fgene.2019.01117)
Supplement: Supplementary file 1 [file Image_1.pdf]

A

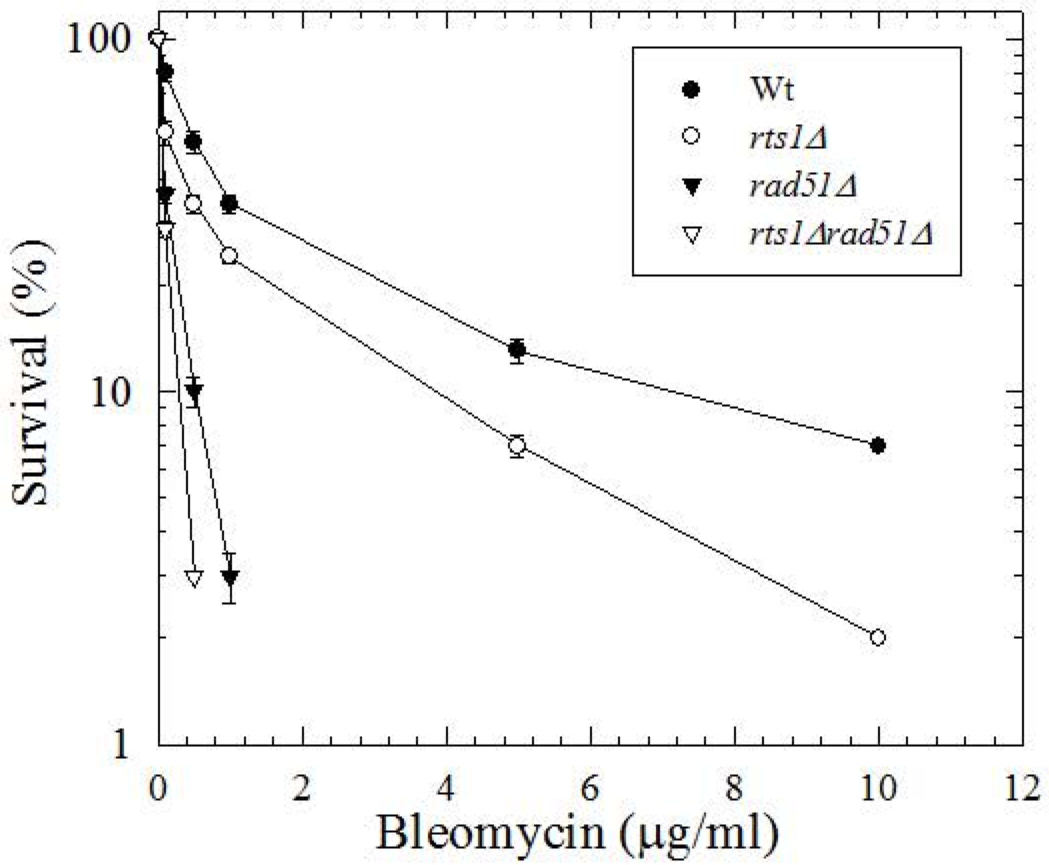

Figure S1A: Survival curves of cells treated in liquid cultures with bleomycin. Survival analysis for bleomycin was performed by treating cells in liquid YPD for 1 hour with the indicated concentration of bleomycin, cells were serially diluted, plated onto YPD solid media and scored for the surviving fractions after 48 hours of growth at 30 °C.

B

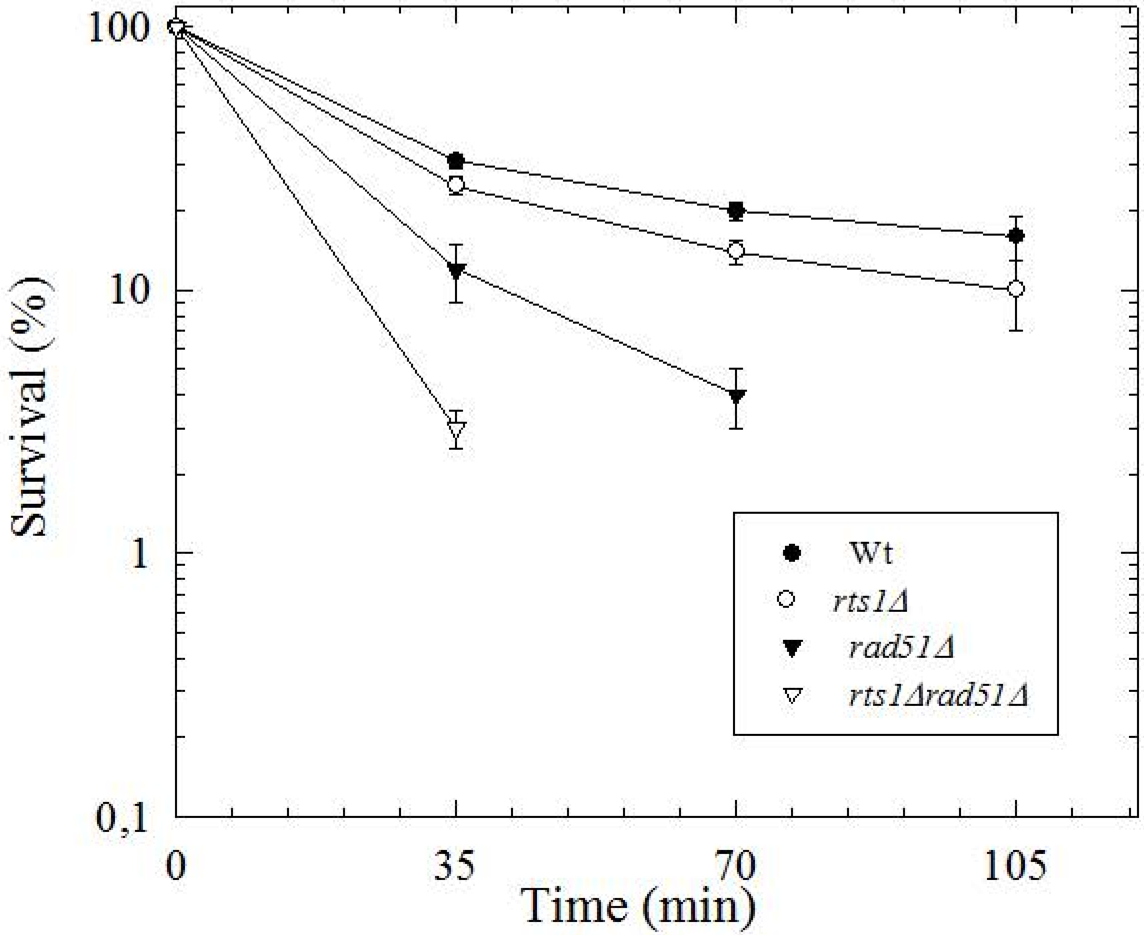

Figure S1B: Survival curves of cells treated in liquid cultures with bleomycin. Cells were treated with a fix concentration of bleomycin (1 µg/ml) for the indicated time before scoring for surviving fractions as in Figure S1A. The data represent the averages of three independent experiments
