# Supplementary figures and images for: Yeast Lacking the PP2A Phosphatase Regulatory Subunit Rts1 Sensitizes rad51 Mutants to Specific DNA Damaging Agents
Source: Front Genet. 2019 Nov 8;10:1117. doi: 10.3389/fgene.2019.01117 (PMC6857479; doi:10.3389/fgene.2019.01117)

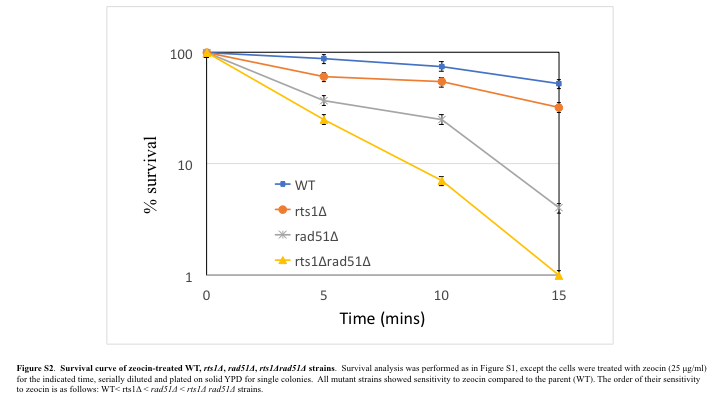

Supplement: Supplementary file 2 [file Image_2.tiff]
